# Changes in Distribution of Severe Neurologic Involvement in US Pediatric Inpatients With COVID-19 or Multisystem Inflammatory Syndrome in Children in 2021 vs 2020

**DOI:** 10.1001/jamaneurol.2022.3881

**Published:** 2022-11-07

**Authors:** Kerri L. LaRovere, Tina Y. Poussaint, Cameron C. Young, Margaret M. Newhams, Suden Kucukak, Katherine Irby, Michele Kong, Stephanie P. Schwartz, Tracie C. Walker, Melania M. Bembea, Kari Wellnitz, Kevin M. Havlin, Natalie Z. Cvijanovich, Mark W. Hall, Julie C. Fitzgerald, Jennifer E. Schuster, Charlotte V. Hobbs, Natasha B. Halasa, Aalok R. Singh, Elizabeth H. Mack, Tamara T. Bradford, Shira J. Gertz, Adam J. Schwarz, Katri V. Typpo, Laura L. Loftis, John S. Giuliano, Steven M. Horwitz, Katherine V. Biagas, Katharine N. Clouser, Courtney M. Rowan, Aline B. Maddux, Vijaya L. Soma, Christopher J. Babbitt, Cassyanne L. Aguiar, Amanda R. Kolmar, Sabrina M. Heidemann, Helen Harvey, Laura D. Zambrano, Angela P. Campbell, Adrienne G. Randolph

**Affiliations:** 1 Department of Neurology, Boston Children’s Hospital, Boston, Massachusetts; 2 Department of Radiology, Boston Children’s Hospital, Boston, Massachusetts; 3 Division of Critical Care Medicine, Department of Anesthesiology, Critical Care and Pain Medicine, Boston Children’s Hospital, Boston, Massachusetts; 4 Section of Pediatric Critical Care, Department of Pediatrics, Arkansas Children's Hospital, Little Rock; 5 Division of Pediatric Critical Care Medicine, Department of Pediatrics, University of Alabama at Birmingham; 6 Department of Pediatrics, University of North Carolina at Chapel Hill Children’s Hospital, Chapel Hill; 7 Division of Pediatric Anesthesiology and Critical Care Medicine, Department of Anesthesiology and Critical Care Medicine, Johns Hopkins School of Medicine, Baltimore, Maryland; 8 Division of Pediatric Critical Care, Stead Family Department of Pediatrics, University of Iowa Carver College of Medicine, Iowa City; 9 Division of Pediatric Critical Care Medicine, Department of Pediatrics, University of Louisville, Norton Children’s Hospital, Louisville, Kentucky; 10 Division of Critical Care Medicine, UCSF Benioff Children’s Hospital, Oakland, California; 11 Division of Critical Care Medicine, Department of Pediatrics, Nationwide Children’s Hospital, Columbus, Ohio; 12 Division of Critical Care, Department of Anesthesiology and Critical Care, University of Pennsylvania Perelman School of Medicine, Philadelphia; 13 Division of Pediatric Infectious Diseases, Department of Pediatrics, Children’s Mercy Kansas City, Kansas City, Missouri; 14 Division of Infectious Diseases, Departments of Pediatrics and Microbiology, University of Mississippi Medical Center, Jackson; 15 Division of Pediatric Infectious Diseases, Department of Pediatrics, Vanderbilt University Medical Center, Nashville, Tennessee; 16 Pediatric Critical Care Division, Maria Fareri Children’s Hospital at Westchester Medical Center, New York Medical College, Valhalla; 17 Division of Pediatric Critical Care Medicine, Medical University of South Carolina, Charleston; 18 Division of Cardiology, Department of Pediatrics, Louisiana State University Health Sciences Center, Children’s Hospital of New Orleans, New Orleans; 19 Division of Pediatric Critical Care, Department of Pediatrics, Cooperman Barnabas Medical Center, Livingston, New Jersey; 20 Division of Critical Care Medicine, Children’s Health Orange County (CHOC), Orange, California; 21 Department of Pediatrics and Banner Children’s at Diamond Children’s Medical Center, University of Arizona, Tucson; 22 Section of Critical Care Medicine, Department of Pediatrics, Texas Children’s Hospital, Houston; 23 Division of Critical Care, Department of Pediatrics, Yale University School of Medicine, New Haven, Connecticut; 24 Division of Pediatric Critical Care Medicine, Department of Pediatrics, Rutgers Robert Wood Johnson Medical School, New Brunswick, New Jersey; 25 Department of Pediatrics, Stony Brook University Renaissance School of Medicine, Stony Brook, New York; 26 Department of Pediatrics, Joseph M. Sanzari Children’s Hospital at Hackensack University Medical Center, Hackensack, New Jersey; 27 Division of Pediatric Critical Care Medicine, Department of Pediatrics, Indiana University School of Medicine, Riley Hospital for Children, Indianapolis; 28 Section of Critical Care Medicine, Department of Pediatrics, University of Colorado School of Medicine and Children’s Hospital Colorado, Aurora; 29 Division of Pediatric Infectious Diseases, Department of Pediatrics, New York University Grossman School of Medicine, New York; 30 Miller Children’s and Women’s Hospital of Long Beach, Long Beach, California; 31 Division of Pediatric Rheumatology, Department of Pediatrics, Eastern Virginia Medical School, Children’s Hospital of The King’s Daughters, Norfolk; 32 Division of Critical Care, Department of Pediatrics, Washington University School of Medicine in St Louis, St Louis, Missouri; 33 Division of Pediatric Critical Care Medicine, Department of Pediatrics, Central Michigan University, Detroit; 34 Division of Pediatric Critical Care, Rady Children’s Hospital, San Diego, California; 35 COVID-19 Response, Centers for Disease Control and Prevention, Atlanta, Georgia; 36 Departments of Anaesthesia and Pediatrics, Harvard Medical School, Boston, Massachusetts

## Abstract

**Importance:**

In 2020 during the COVID-19 pandemic, neurologic involvement was common in children and adolescents hospitalized in the United States for severe acute respiratory syndrome coronavirus 2 (SARS-CoV-2)–related complications.

**Objective:**

To provide an update on the spectrum of SARS-CoV-2–related neurologic involvement among children and adolescents in 2021.

**Design, Setting, and Participants:**

Case series investigation of patients reported to public health surveillance hospitalized with SARS-CoV-2–related illness between December 15, 2020, and December 31, 2021, in 55 US hospitals in 31 states with follow-up at hospital discharge. A total of 2253 patients were enrolled during the investigation period. Patients suspected of having multisystem inflammatory syndrome in children (MIS-C) who did not meet criteria (n = 85) were excluded. Patients (<21 years) with positive SARS-CoV-2 test results (reverse transcriptase–polymerase chain reaction and/or antibody) meeting criteria for MIS-C or acute COVID-19 were included in the analysis.

**Exposure:**

SARS-CoV-2 infection.

**Main Outcomes and Measures:**

Patients with neurologic involvement had acute neurologic signs, symptoms, or diseases on presentation or during hospitalization. Life-threatening neurologic involvement was adjudicated by experts based on clinical and/or neuroradiological features. Type and severity of neurologic involvement, laboratory and imaging data, vaccination status, and hospital discharge outcomes (death or survival with new neurologic deficits).

**Results:**

Of 2168 patients included (58% male; median age, 10.3 years), 1435 (66%) met criteria for MIS-C, and 476 (22%) had documented neurologic involvement. Patients with neurologic involvement vs without were older (median age, 12 vs 10 years) and more frequently had underlying neurologic disorders (107 of 476 [22%] vs 240 of 1692 [14%]). Among those with neurologic involvement, 42 (9%) developed acute SARS-CoV-2–related life-threatening conditions, including central nervous system infection/demyelination (n = 23; 15 with possible/confirmed encephalitis, 6 meningitis, 1 transverse myelitis, 1 nonhemorrhagic leukoencephalopathy), stroke (n = 11), severe encephalopathy (n = 5), acute fulminant cerebral edema (n = 2), and Guillain-Barré syndrome (n = 1). Ten of 42 (24%) survived with new neurologic deficits at discharge and 8 (19%) died. Among patients with life-threatening neurologic conditions, 15 of 16 vaccine-eligible patients (94%) were unvaccinated.

**Conclusions and Relevance:**

SARS-CoV-2–related neurologic involvement persisted in US children and adolescents hospitalized for COVID-19 or MIS-C in 2021 and was again mostly transient. Central nervous system infection/demyelination accounted for a higher proportion of life-threatening conditions, and most vaccine-eligible patients were unvaccinated. COVID-19 vaccination may prevent some SARS-CoV-2–related neurologic complications and merits further study.

## Key Points

**Question:**

What was the spectrum of SARS-CoV-2–related pediatric severe neurologic involvement in 2021?

**Findings:**

In this case series of 2168 US patients younger than 21 years hospitalized for acute COVID-19 (34%) or multisystem inflammatory syndrome in children (66%), 476 (22%) had neurologic involvement. Of these, 42 (9%) had life-threatening conditions, with 23 (55%) having acute central nervous system (CNS) infections/demyelination; 18 of 42 (43%) died or had new neurologic deficits; and most vaccine-eligible patients were unvaccinated.

**Meaning:**

In 2021, SARS-CoV-2–related severe neurologic involvement in US hospitalized children and adolescents showed a potential increase in diagnoses of acute CNS infections/demyelination.

## Teaser

This case series assesses data from 55 US hospitals about neurologic involvement in children and adolescents with acute COVID-19 or multisystem inflammatory syndrome in children.

## Introduction

In 2020, neurologic involvement in severe acute COVID-19 or multisystem inflammatory syndrome in children (MIS-C) was identified in 22% of pediatric patients (365 of 1695) hospitalized at 52 US sites from March to mid-December, and 12% had severe complications.^1^ In June 2021, the B.1.617.2 (Delta) variant of SARS-CoV-2 became predominant, causing another surge of US pediatric hospitalizations.^2^ In 2021, children became eligible for COVID-19 vaccination (May 12, 2021, for adolescents,^3^ November 2, 2021, for children aged 5-11 years^4^). This update on the extent of SARS-CoV-2–related neurologic involvement and documented hospital outcomes in US children and adolescents evaluates patients hospitalized during 2021 including their COVID-19 vaccination status. Data for the update came from active surveillance performed at hospitals participating in the Overcoming COVID-19 public health surveillance network (eAppendix in Supplement 1).

## Methods

### Design and Participants

We performed active surveillance at 55 hospitals in 31 states to identify US patients (<21 years) with severe acute COVID-19 (admitted to an intensive care or step-down unit at a participating site) or who met Centers for Disease Control and Prevention (CDC) criteria for MIS-C hospitalized between December 15, 2020, and December 31, 2021 (eTable 1 in Supplement 1). Patients with acute COVID-19 had a positive result on a SARS-CoV-2 respiratory test (reverse transcriptase–polymerase chain reaction [RT-PCR] or antigen) and symptoms related to COVID-19. Patients with MIS-C had a positive SARS-CoV-2 respiratory or antibody test result. Reporting guidelines for uncontrolled case series were followed.^5^ The investigation was approved by the central institutional review board at Boston Children’s Hospital and determined to meet the requirement of public health surveillance as defined in 45 CFR 46.102(I)(2) by the CDC with waiver of consent.

### Classification of Neurologic Involvement and Outcomes

Data were abstracted from medical records by trained staff.^1^ Patients with life-threatening neurologic conditions and neurologic deficits (gross impairment in motor, cognitive, or speech and language functions) identified from medical records were adjudicated by neurology, neuroradiology, and critical care experts (K.L.L., T.Y.P., A.G.R.). Cases of encephalitis were adjudicated using standardized case report forms including the International Encephalitis Consortium criteria.^6^ Race and ethnicity were extracted from medical records, and social vulnerability index^7^ was calculated from home addresses. Vaccination eligibility and status were confirmed as previously reported (eMethods in Supplement 1).

### Statistical Analyses

Descriptive statistics were used to report frequencies. Continuous variables included median and IQR, and categorical variables included counts and percentages. We used a χ^2^ test, Fisher exact test, or Kruskal-Wallis test to evaluate between-group differences using R (version 4.0.2, R Project for Statistical Computing) with *P* < .05 considered statistically significant. Missing data were not imputed.

## Results

Of 2168 patients (58% male; median age, 10.3 years) with acute COVID-19 (34%) or MIS-C (66%), 476 (22%) had neurologic involvement (Table 1 and eFigure 1 in Supplement 1). Patients with neurologic involvement were older and had more underlying neurologic disorders than those without. Seizures were more common in younger children, and loss of taste and smell was more common in adolescents (eTable 2 and eFigure 2 in Supplement 1).

**Table 1.** Characteristics and Outcomes of 2168 Patients (<21 Years) Hospitalized for SARS-CoV-2–Related Illness by Reported Neurologic Involvement During the Second Year of the Pandemic (December 15, 2020, to December 31, 2021)

Characteristic	No. (%)			*P* value
	All patients	With neurological involvement	Without neurological involvement	
No.	2168	476	1692	
Sex				.93
Male	1260 (58)	278 (58)	982 (58)	
Female	908 (42)	198 (42)	710 (42)	
Age, y				<.001
Median (IQR)	10.3 (5.9-14.7)	11.7 (7.1-15.8)	10.0 (5.5-14.3)	
<1	120 (6)	32 (7)	88 (5)	
1-<5	342 (16)	44 (9)	298 (18)	
5-<12	832 (38)	172 (36)	660 (39)	
12-<18	783 (36)	202 (42)	581 (34)	
18-<21	91 (4)	26 (5)	65 (4)	
Race and ethnic group^a^				.19
Asian	58 (3)	12 (3)	46 (3)	
Hispanic or Latino	467 (22)	93 (20)	374 (22)	
Non-Hispanic Black	621 (29)	150 (32)	471 (28)	
Non-Hispanic White	778 (36)	180 (38)	598 (35)	
Other race, non-Hispanic	52 (2)	8 (2)	44 (3)	
Unknown	192 (9)	33 (7)	159 (9)	
Socioeconomic status				.08
Government/public insurance	1240 (57)	290 (61)	950 (56)	
Private/self-pay	869 (40)	169 (36)	700 (41)	
Unknown insurance	59 (3)	15 (3)	44 (3)	
SVI, median (IQR)	0.59 (0.32-0.82)	0.65 (0.37-0.81)	0.58 (0.30-0.82)	.08
Lowest third (least vulnerable)	534 (25)	99 (21)	453 (26)	.05
Middle third	659 (30)	140 (29)	519 (31)	
Highest third (most vulnerable)	876 (40)	211 (44)	665 (39)	
Missing SVI	99 (5)	26 (6)	73 (4)	
SARS-CoV-2 testing				
RT-PCR performed	2017 (93)	458 (96)	1559 (92)	.003
RT-PCR positive	1153/2017 (57)	304/458 (66)	849/1559 (54)	<.001
Antibody test performed	1539 (71)	303 (64)	1236 (73)	<.001
Antibody test result positive	1442/1539 (94)	275/303 (91)	1167/1236 (94)	.03
Date of admission				
December 2020-May 2021 (pre-Delta)	1096 (51)	217 (46)	879 (52)	.02
June 2021-December 2021 (Delta)	1072 (49)	259 (54)	813 (48)	
Underlying conditions^b^				
Previously healthy^c^	1128 (52)	204 (43)	924 (55)	<.001
≥1 Comorbidity, excluding obesity	788 (36)	216 (45)	572 (34)	<.001
Neurological, any condition	347 (16)	107 (22)	240 (14)	<.001
Neurodevelopmental/psychiatric^d^	274 (13)	79 (17)	195 (12)	.004
Seizure disorder	114 (5)	51 (11)	63 (4)	<.001
Neuromuscular disorders^e^	104 (5)	40 (8)	64 (4)	<.001
Congenital neurologic disorders^f^	30 (1)	18 (4)	12 (0.7)	<.001
Static encephalopathy	26 (1)	10 (2)	16 (0.9)	.07
Other^g^	11 (0.5)	3 (0.6)	8 (0.5)	.72
Respiratory	428 (20)	99 (21)	329 (19)	.56
Gastrointestinal	167 (8)	52 (11)	115 (7)	.004
Endocrine	129 (6)	55 (12)	74 (4)	<.001
Cardiac	104 (5)	30 (6)	74 (4)	.11
Genetic or metabolic (not obesity)	81 (4)	27 (6)	54 (3)	.02
Hematological	71 (3)	16 (3)	55 (3)	>.99
Oncologic or immune compromised	64 (3)	14 (3)	50 (3)	>.99
Renal	58 (3)	22 (5)	36 (2)	.01
BMI-based obesity^h^	708 (36)	168 (39)	540 (35)	.12
Organ system involvement				
Met MIS-C criteria	1435 (66)	269 (57)	1166 (69)	<.001
Organ systems involved, median (IQR)	4 (3-5)	5 (3-6)	3 (2-4)	<.001
Vaccination status^i^				
Vaccine eligible	664 (31)	185 (39)	479 (28)	<.001
Fully	10 (2)	4 (2)	6 (1)	.53
Partially	17 (3)	4 (2)	13 (3)	
Unvaccinated	511 (77)	147 (79)	364 (76)	
Unknown vaccination status	126 (19)	30 (16)	96 (20)	
Outcomes				
ICU admission	1560 (72)	400 (84)	1160 (69)	<.001
ECMO	113 (5)	46 (10)	67 (4)	<.001
Mechanical ventilation	336 (16)	146 (31)	190 (11)	<.001
Vasopressors	768 (35)	226 (48)	542 (32)	<.001
Length of stay, median (IQR), d				
ICU	3 (2-6)	5 (2-10)	3 (2-6)	<.001
Hospital	6 (4-9)	7 (5-14)	5 (4-8)	<.001
Died	56 (3)	31 (7)	26 (2)	<.001
Survived, new neurological deficit	39 (2)	29 (6)	9 (0.5)	<.001
Discharged to rehabilitation/other acute care facility	51 (2)	23 (5)	28 (2)	<.001

Abbreviations: BMI, body mass index; CNS, central nervous system; ECMO, extracorporeal membrane oxygenation; HIE, hypoxic ischemic encephalopathy; ICU, intensive care unit; MIS-C, multisystem inflammatory syndrome in children; RT-PCR, reverse transcriptase–polymerase chain reaction; SVI, social vulnerability index^5^ (higher scores indicate higher vulnerability).

^a^ Race and ethnic group were abstracted from the patient’s medical record. Race categories are not mutually exclusive.

^b^ Patients may have more than 1 underlying condition.

^c^ “Previously healthy” was defined as an absence of reported underlying conditions (including obesity) and taking no prescription medications.

^d^ Neurodevelopmental/psychiatric conditions include developmental delay, cognitive delay, gross motor delay, and intellectual disability; psychiatric disorders include attention-deficit/hyperactivity disorder, mood disorder, and autism/autism spectrum disorder.

^e^ Neuromuscular disorders include spastic quadriplegia, muscular dystrophy, neuromuscular weakness, and neuromuscular scoliosis.

^f^ Congenital neurologic disorders include hydrocephalus, neurogenetic, and neurometabolic disorders.

^g^ Other underlying neurological conditions include history of CNS tumor or traumatic brain injury, prior stroke/hypoxic ischemic injury, and history of CNS infection/demyelinating disorder.

^h^ The determination of BMI-based obesity was based on the Centers for Disease Control and Prevention national reference standard for age and sex among patients who were at least 2 years of age (n = 426 for patients with neurological involvements and n = 1534 for patients without neurological involvement).

^i^ Vaccination eligibility/status was defined as previously reported (eMethods in Supplement 1).

In patients with neurologic involvement, 91% had non–life-threatening neurologic symptoms, most commonly fatigue/weakness, confusion, headache, and loss of taste/smell. Among patients with non–life-threatening neurologic involvement, 90% survived without neurologic deficits, 5% died, and 4% were discharged alive with neurologic deficits related to sequelae of critical illness. A spectrum of life-threatening neurologic conditions and outcomes were identified in 42 of 476 patients (9%) with neurologic involvement, including 23 (55%) with acute central nervous system (CNS) infection/acute disseminated encephalomyelitis (ADEM). Life-threatening neurologic conditions were more frequently reported during the Delta than pre-Delta periods (64% vs 36%). Ten of 42 patients (24%) survived with new neurologic deficits at discharge and 8 (19%) died (Table 2 and eTable 3 in Supplement 1).

**Table 2.** Life-threatening Neurologic Conditions and Deaths Related to COVID-19 or MIS-C in 42 Hospitalized Patients (<21 Years) During the Second Year of the Pandemic (December 15, 2020, to December 31, 2021)

Variable	Life-threatening SARS-CoV-2–related neurologic conditions, No. (%)					
	Any condition	Acute CNS infection or ADEM	Ischemic or hemorrhagic stroke	Severe encephalopathy	Acute fulminant cerebral edema	Guillain-Barré Syndrome
No.	42	23	11	5	2	1
Age, median (IQR), y^a^	11 (6-16)	1 Infant	2 Infants	1 Infant	2 Teenagers	1 School age
		1 Toddler	2 Toddlers	1 School age		
		2 Preschoolers	1 Preschooler	3 Teenagers		
		9 School age	3 School age			
		9 Teenagers	3 Teenagers			
		1 Young adult				
Male sex	29 (67)	13 (57)	10 (91)	4 (80)	2 (100)	0
SARS-CoV-2 strain						
Pre-Delta (December 15, 2020, to May 2021)	15 (36)	9 (39)	4 (36)	2 (40)	0	0
Delta (June 2021 to December 31, 2021)	27 (64)	14 (61)	7 (64)	3 (60)	2 (100)	1 (100)
RT-PCR or antibody results						
RT-PCR positive only	19 (45)	9 (39)	7 (64)	1 (20)	1 (50)	1 (100)
Antibody positive only	13 (31)	8 (35)	1 (9)	3 (60)	1 (50)	0
RT-PCR and antibody positive	10 (24)	6 (26)	3 (27)	1 (20)	0	0
MIS-C diagnosis	20 (48)	13 (57)	3 (27)	3 (60)	1 (50)	0
No major underlying conditions	16 (38)	8 (35)	5 (45)	2 (40)	0	1 (100)
Underlying neurologic disorder	5 (12)	1 (4)	3 (27)	0	1 (50)	0
Death	7 (17)	3 (13)	3 (27)	0	2 (100)	0
Discharged alive, new CNS deficit	11 (26)	4 (17)	5 (45)	0	0	1 (100)
Vaccine eligible	16 (38)	11 (48)	2 (18)	1 (20)	2 (100)	0
Fully or partially vaccinated/eligible	1/16 (6)	1/11 (9)	0	0	0	0

Abbreviations: ADEM, acute disseminated encephalomyelitis; CNS, central nervous system; MIS-C, multisystem inflammatory syndrome in children; RT-PCR, reverse transcriptase–polymerase chain reaction.

^a^ Age categories reported for privacy reasons for subcategories of neurologic conditions are infant (<1 year), toddler (1-2 years), preschooler (3-5 years), school age (6-12 years), teenager (13-17 years), and young adult (18-21 years).

There were 9 possible and 5 confirmed cases of encephalitis (eTable 4 in Supplement 1). Electroencephalography abnormalities included diffuse background slowing (n = 10) and/or focal seizures or epileptic discharges (n = 5). Brain magnetic resonance imaging (MRI) findings were mostly ADEM-like with multifocal, nonenhancing lesions with T2 prolongation and reduced diffusivity mainly in the deep juxtacortical and periventricular white matter, thalami, basal ganglia, brainstem, and posterior fossa and in 1 case cortical involvement in the supratentorium (eTable 4 in Supplement 1). One patient had low titer–positive myelin oligodendrocyte glycoprotein antibody (1:20) with involvement of the left temporal lobe on MRI that resolved on 9-month follow-up brain MRI. Of 23 patients with acute CNS infection/ADEM, outcomes were severe in 7 patients (30%) (Table 2 and eTable 1 in Supplement 1). Representative brain MRI studies from 2 patients with acute encephalitis and 1 with meningitis and cerebral venous sinus thrombosis complication are shown in the Figure.

**Figure.**
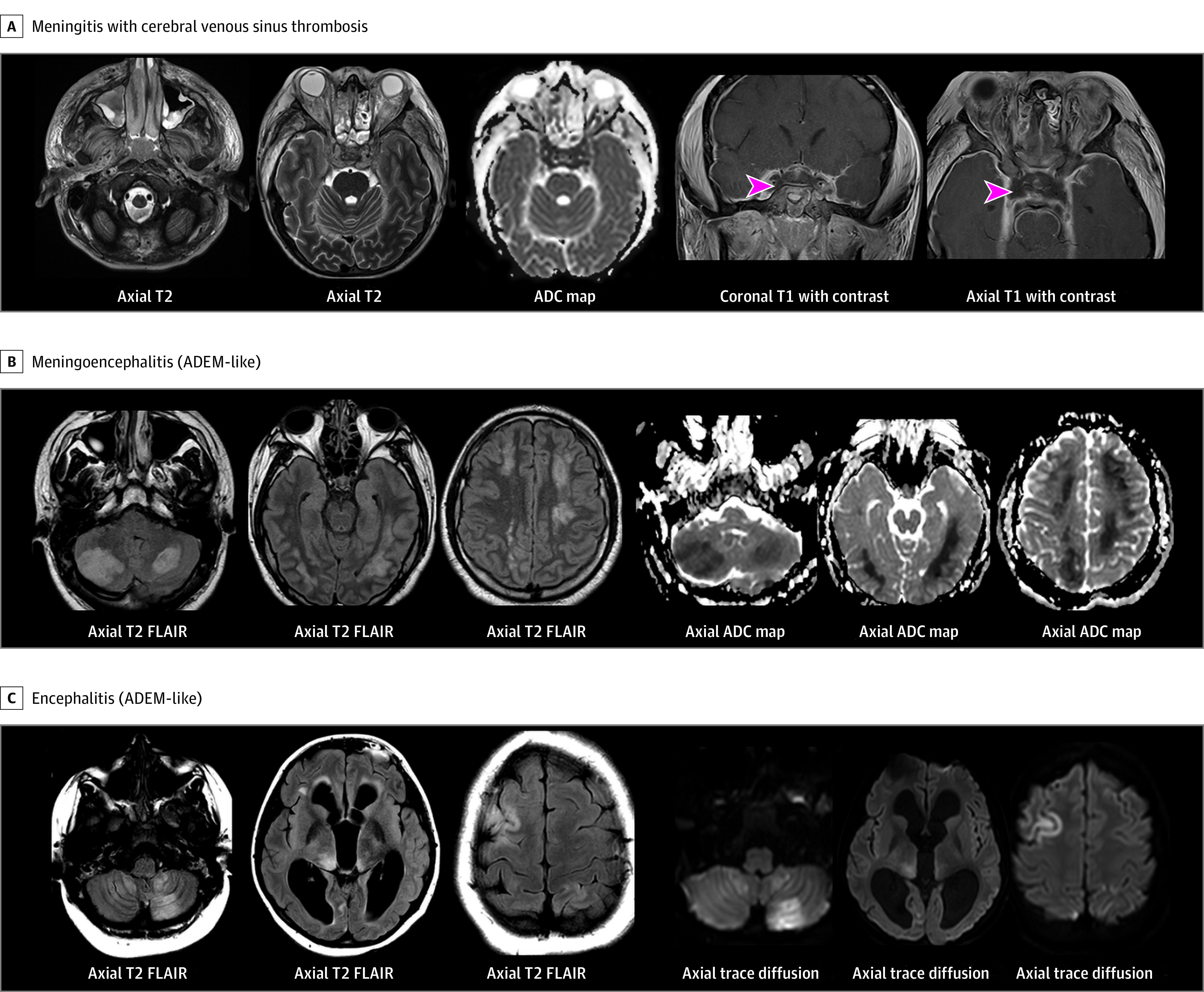
Representative Central Nervous System Images From Patients With Life-threatening COVID-19–Related Neurologic Involvement A, Teenager presented with acute respiratory failure, fever, acute onset of altered awareness, confusion, agitation, and difficulty walking. Axial T2 images show myositis of the facial muscles with stranding of subcutaneous fat, sinusitis, and cavernous sinus thrombosis with T2 hypointense signal and reduced diffusivity (apparent diffusion coefficient [ADC] map). Coronal and axial T1 images with contrast demonstrate dural enhancement, filling defects in superior ophthalmic veins and cavernous sinuses consistent with thrombosis (arrowheads). The patient’s brain injury evolved to diagnosis of death by neurologic criteria. B, Teenager with history of obesity, hypertension, and diabetes presented with 3 weeks of fever, confusion, headache, seizures, orofacial dyskinesias, agitation, slurred speech, difficulty walking, chorea, and left-sided weakness. Axial T2 fluid-attenuated inversion recovery (FLAIR) images demonstrate T2 hyperintense signal in bilateral cerebellar, bilateral temporal, and bilateral centrum semiovale white matter with reduced diffusivity. There was no enhancement, and susceptibility was present in lesions consistent with punctate blood products (not shown). The patient later died by brain death. C, Teenager presented with 3 weeks of fever, headaches, lethargy, confusion, seizure, vomiting, blurry vision, and nystagmus. Axial T2 FLAIR images demonstrate moderate enlargement of lateral and third ventricles, T2 prolongation in bilateral cerebellar hemispheres, bilateral thalami, and bifrontal white matter and cortex with reduced diffusivity on trace diffusion images and no enhancement (not shown). ADEM indicates acute disseminated encephalomyelitis.

Of the 155 vaccine-eligible patients with neurologic involvement and confirmed vaccination status, 147 (95%) were unvaccinated (Table 1 and eTable 5 in Supplement 1), including 15 of 16 patients (94%) with life-threatening neurologic conditions (Table 2).

## Discussion

In 2168 US children and adolescents hospitalized with acute COVID-19 or MIS-C during 2021, the frequency, range, and severity of neurologic involvement were similar to the 2020 investigation.^1^ However, there were 2 major differences between the 2 surveillance periods. First, acute CNS infection/ADEM cases accounted for a higher proportion of life-threatening cases (55% in 2021 vs 19% in 2020). Many of these patients had subacute onset of encephalitis-like symptoms and ADEM-like imaging features and were discharged home, but 30% had severe outcomes. Because the adjudication methods for neurologic involvement were the same in both years, it is possible that the increased number of acute CNS infection/ADEM cases in 2021 were associated with the Delta variant^8^ or due to more diagnostic investigations in 2021 identifying more cases. Second, most patients with severe COVID-19 or MIS-C associated neurologic involvement who were vaccine eligible were unvaccinated.

Animal model data for SARS-CoV-2–induced encephalomyelitis support the possibility that it may contribute to the immunopathogenesis of encephalitis in humans.^9^ Brain biopsies in 9 adults with fatal COVID-19 revealed extensive inflammation of both white and gray matter without detectable SARS-CoV-2.^10^ Whether COVID-19 vaccination can prevent SARS-CoV-2–associated neurologic complications merits further study, weighing the immune-mediated vaccine-specific adverse neurologic events.^11,12^

### Limitations

This investigation has multiple limitations. As a public health surveillance investigation, we were unable to study the immunobiology underlying severe complications, including the Delta variant, or assess reasons for low vaccine uptake. It is probable that we did not capture all patients. Fatigue, weakness, and headache are nonspecific symptoms that could lead to overreporting of milder neurologic involvement. Although data collection was standardized, potential misclassification of patients with neurologic involvement, including acute CNS infection/ADEM, may have occurred because of nonstandardized diagnostic investigations at each site, conducted using clinical discretion. ADEM was categorized based on the presence of encephalopathy and acute imaging features only and therefore is unconfirmed based on the 2013 International Pediatric Multiple Sclerosis Society Group criteria.^13^ Myelin oligodendrocyte glycoprotein has been reported in pediatric patients with COVID-19,^14^ but we cannot determine in the 1 patient if it represents a primary or autoimmune response to SARS-CoV-2 or is unrelated.

## Conclusions

SARS-CoV-2–related neurologic involvement in US children and adolescents hospitalized for COVID-19 or MIS-C persisted in 2021, and acute CNS infection/ADEM accounted for more of the reported life-threatening cases than in 2020. COVID-19 vaccination became available for adolescents and children during 2021, but most vaccine-eligible patients were unvaccinated. COVID-19 vaccination is effective at preventing hospitalization for acute COVID-19^15^ and MIS-C^16^ and may decrease associated neurologic complications.

## Notes

Supplement 1. **eAppendix.** Overcoming COVID-19 Investigators and CDC COVID-19 Response Team
eMethods
**eFigure 1.** Eligibility flowchart of hospitalized patients with SARS-CoV-2-related neurologic involvement, December 15, 2020, – December 31, 2022 **eFigure 2.** Presenting neurologic symptoms and most abnormal laboratory values in patients (<21 years) hospitalized for COVID-19 or MIS-C **eTable 1.** Case definition used in this investigation for multisystem inflammatory syndrome in children (MIS-C) **eTable 2.** Number of patients with presenting neurologic symptoms (2A) and number of patients with most abnormal laboratory values by severity of neurologic involvement (2B) **eTable 3.** Life-threatening SARS-CoV-2-related neurologic disorders and outcomes in 42 children and adolescents (<21 years) hospitalized for COVID-19 or MIS-C **eTable 4.** Detailed clinical descriptions, treatment and outcomes for 14 patients (<21 years) with clinically adjudicated possible/confirmed acute encephalitis **eTable 5.** Vaccination status by SARS-CoV-2-related neurologic involvement and MIS-C versus acute COVID-19 diagnosis
eReferences

Supplement 2. Nonauthor collaborators
