# Supplementary material for: Changes in Distribution of Severe Neurologic Involvement in US Pediatric Inpatients With COVID-19 or Multisystem Inflammatory Syndrome in Children in 2021 vs 2020
Source: JAMA Neurol. 2022 Nov 7;80(1):91–8. doi: 10.1001/jamaneurol.2022.3881 (PMC9641594; doi:10.1001/jamaneurol.2022.3881)
Supplement: Supplement 1. — eAppendix. Overcoming COVID-19 Investigators and CDC COVID-19 Response Team eMethods eFigure 1. Eligibility flowchart of hospitalized patients with SARS-CoV-2-related neurologic involvement, December 15, 2020, – December 31, 2022 eFigure 2. Presenting neurologic symptoms and most abnormal laboratory values in patients (<21 years) hospitalized for COVID-19 or MIS-C eTable 1. Case definition used in this investigation for multisystem inflammatory syndrome in children (MIS-C) eTable 2. Number of patients with presenting neurologic symptoms (2A) and number of patients with most abnormal laboratory values by severity of neurologic involvement (2B) eTable 3. Life-threatening SARS-CoV-2-related neurologic disorders and outcomes in 42 children and adolescents (<21 years) hospitalized for COVID-19 or MIS-C eTable 4. Detailed clinical descriptions, treatment and outcomes for 14 patients (<21 years) with clinically adjudicated possible/confirmed acute encephalitis eTable 5. Vaccination status by SARS-CoV-2-related neurologic involvement and MIS-C versus acute COVID-19 diagnosis eReferences [file jamaneurol-e223881-s001.pdf]

Supplemental Online Content

LaRovere KL, Poussaint TY, Young CC, et al; the Overcoming COVID-19 Investigators. Changes in distribution of severe neurologic involvement in US pediatric inpatients with COVID-19 or multisystem inflammatory syndrome in children in 2021 vs 2020. *JAMA Neurol*. Published online November 7, 2022. doi:10.1001/jamaneurol.2022.3881

eAppendix. Overcoming COVID-19 Investigators and CDC COVID-19 Response Team

eMethods

eFigure 1. Eligibility flowchart of hospitalized patients with SARS-CoV-2-related neurologic involvement, December 15, 2020, – December 31, 2022

eFigure 2. Presenting neurologic symptoms and most abnormal laboratory values in patients (<21 years) hospitalized for COVID-19 or MIS-C

eTable 1. Case definition used in this investigation for multisystem inflammatory syndrome in children (MIS-C)

eTable 2. Number of patients with presenting neurologic symptoms (2A) and number of patients with most abnormal laboratory values by severity of neurologic involvement (2B)

eTable 3. Life-threatening SARS-CoV-2-related neurologic disorders and outcomes in 42 children and adolescents (<21 years) hospitalized for COVID-19 or MIS-C

eTable 4. Detailed clinical descriptions, treatment and outcomes for 14 patients (<21 years) with clinically adjudicated possible/confirmed acute encephalitis

eTable 5. Vaccination status by SARS-CoV-2-related neurologic involvement and MIS-C versus acute COVID-19 diagnosis

eReferences

This supplemental material has been provided by the authors to give readers additional information about their work.

© 2022 LaRovere KL et al. *JAMA Neurology.*

eAppendix. Overcoming COVID-19 Investigators
(listed in PubMed, and ordered by U.S. State)

The following investigators were all closely involved with the design, implementation, and oversight of the Overcoming COVID-19 public health surveillance investigation.

Alabama: Children's of Alabama, Birmingham. Michele Kong, MD.

Arizona: University of Arizona, Tucson. Mary Glas Gaspers, MD; Katri V. Typpo, MD.

Arkansas: Arkansas Children's Hospital, Little Rock. Ronald C. Sanders, MD, MS; Katherine Irby, MD.

California: Children's Hospital of Orange County, Orange County. Adam J. Schwarz, MD.

California: Miller Children's & Women's Hospital Long Beach, Long Beach. Christopher J. Babbitt, MD.

California: UCSF Benioff Children's Hospital Oakland, Oakland. Natalie Z. Cvijanovich, MD.

California: UCSF Benioff Children's Hospital, San Francisco. Matt S. Zinter, MD

California: Rady Children's Hospital, San Diego. Helen Harvey, MD, MS.

California: Children's Hospital Los Angeles, Los Angeles. Pia S. Pannaraj, MD, MPH.

Colorado: Children's Hospital Colorado, Aurora. Aline B. Maddux, MD, MSCS; Emily Port, BA, PMP; Sara Shankman, DNP, CPNC-AC; Rachel Mansour, BSN, RN, CPN.

Connecticut: Connecticut Children's, Hartford. Christopher L. Carroll, MD, MS.

Connecticut: Yale New-Haven Children's Hospital, New Haven. John S. Giuliano, Jr., MD.

Florida: Holtz Children's Hospital, Miami. Gwenn E. McLaughlin, MD, MSPH.

Florida: Nicklaus Children's Hospital, Miami. Paula S. Espinal, MD, MPH.

Illinois: Ann & Robert H. Lurie Children's Hospital of Chicago, Chicago. Kelly N. Michelson, MD, MPH; Bria M. Coates, MD.

Indiana: Riley Hospital for Children, Indianapolis. Courtney M. Rowan, MD, MS.

Iowa: University of Iowa Stead Family Children's Hospital, Iowa City. Kari Wellnitz, MD; Guru Bhoojhawon MBBS, MD.

Kentucky: University of Louisville and Norton Children's Hospital, Louisville. Janice E. Sullivan, MD; Vicki L. Montgomery, MD; Kevin M. Havlin, MD.

Louisiana: Children's Hospital of New Orleans, New Orleans. Tamara T. Bradford, MD.

Maryland: Johns Hopkins Children's Hospital, Baltimore. Melania M. Bembea, MD, MPH, PhD.

Maryland: University of Maryland Children's Hospital, Baltimore. Ana Lia Graciano, MD.

Maryland: Sinai Hospital of Baltimore, Baltimore. Susan V. Lipton, MD, MPH.

Massachusetts: Boston Children's Hospital, Boston. Adrienne G. Randolph, MD; Margaret M. Newhams, MPH; Cameron C. Young; Kerri L. LaRovere, MD; Tina Y. Poussaint, MD; Mary Beth F. Son, MD; Suden Kucukak, MD; Sabrina R. Chen; Julia Worden; Timothy McCadden.

Massachusetts: MassGeneral Hospital for Children, Boston. Ryan W. Carroll, MD, MPH; Phoebe H. Yager, MD; Neil D. Fernandes, MBBS.

Michigan: Children's Hospital of Michigan, Detroit. Sabrina M. Heidemann, MD.

© 2022 LaRovere KL et al. *JAMA Neurology.*

Minnesota: University of Minnesota Masonic Children's Hospital, Minneapolis, Janet R. Hume, MD, PhD.

Minnesota: Mayo Clinic, Rochester. Emily R. Levy, MD.

Mississippi: Children's Hospital of Mississippi, Jackson. Charlotte V. Hobbs, MD; Lacy Malloch, BS; Lora Martin, MSN; Candace Howard-Claudio MD; David Gourdy MD.

Missouri: Children's Mercy Hospital, Kansas City. Jennifer E. Schuster, MD.

Missouri: Washington University in St. Louis. Philip C. Spinella MD; Amanda R. Kolmar MD.

Nebraska: Children's Hospital & Medical Center, Omaha. Melissa L. Cullimore, MD, PhD; Russell J. McCulloh, MD.

New Jersey: Hackensack University Medical Center, Hackensack. Katharine N. Clouser, MD.

New Jersey: Bristol-Myers Squibb Children's Hospital, New Brunswick. Lawrence C. Kleinman, MD, MPH, FAAP; Simon Li, MD, MPH; Steven M. Horwitz, MD.

New Jersey: Cooperman Barnabas Medical Center, Livingston. Shira J. Gertz, MD.

New York: Golisano Children's Hospital, Rochester. Joseph Kuebler, MD.

New York: Maria Fareri Children's Hospital, Valhalla. Aalok R. Singh, MD.

New York: Hassenfeld Children's Hospital at NYU Langone, New York. Adam J. Ratner, MD, MPH; Heda Dapul, MD; Vijaya L. Soma, MD.

New York: Stony Brook University Hospital, Stony Brook. Katherine V. Biagas MD.

New York: SUNY Downstate Medical Center University Hospital, Brooklyn. Sule Doymaz, MD.

New York: Kings County Hospital, New York. Michael A. Keenaghan, MD.

North Carolina: University of North Carolina at Chapel Hill, Chapel Hill. Stephanie P. Schwartz, MD; Tracie C. Walker, MD; Cody Schwartz MD, MPH.

Ohio: University Hospitals Rainbow Babies and Children's Hospital, Cleveland. Steven L. Shein, MD; Amanda N. Lansell, MD.

Ohio: Nationwide Children's Hospital, Columbus. Mark W. Hall MD, FCCM.

Ohio: Akron Children's Hospital, Akron. Ryan A. Nofziger, MD, MBA.

Ohio: Cincinnati Children's Hospital, Cincinnati. Mary A. Staat, MD, MPH.

Pennsylvania: Children's Hospital of Philadelphia, Philadelphia. Julie C. Fitzgerald, MD, PhD, MSCE.

Pennsylvania: Penn State Children's Hospital, Hershey. Neal J. Thomas, MD, MSc.

Pennsylvania: St. Christopher's Hospital for Children, Philadelphia. Andrew Butler MD.

South Carolina: MUSC Shawn Jenkins Children's Hospital, Charleston. Elizabeth H. Mack, MD, MS; Nelson Reed MD.

Tennessee: Monroe Carell Jr. Children's Hospital at Vanderbilt, Nashville. Natasha B. Halasa, MD, MPH; Laura Stewart, PhD; Meena Golchha, MD.

Texas: Texas Children's Hospital, Houston. Laura L. Loftis, MD; Marian Samperio MD.

Texas: University of Texas Southwestern, Children's Medical Center Dallas, Dallas. Cindy Bowens, MD, MSCS; Mia Maamari, MD.

© 2022 LaRovere KL et al. *JAMA Neurology.*

Utah: Primary Children's Hospital, Salt Lake City. Hillary Crandall, MD, PhD.

Virginia: Children's Hospital of The King's Daughters, Norfolk. Cassyanne L. Aguiar, MD.

Washington: Seattle Children's Hospital, Seattle. Lincoln S. Smith, MD; John K. McGuire, MD.

CDC COVID-19 Response Team on Overcoming COVID-19: Angela Campbell, MD, MPH; Laura Zambrano, MPH, PhD; Manish M. Patel, MD, MPH

© 2022 LaRovere KL et al. *JAMA Neurology.*

eMethods

The *Overcoming COVID-19* Public Health Surveillance Registry is funded by the United States Centers for Disease Control and Prevention (CDC) in collaboration with the Pediatric Intensive Care Influenza and Emerging Pathogens (PICFLU-EP) Network which is a subgroup of the Pediatric Acute Lung Injury and Sepsis Investigator's (PALISI) Network. Targeted retrospective and prospective surveillance in children and adolescents for acute COVID-19 and MIS-C has been ongoing since March 15, 2020 at pediatric surveillance sites across the United States. The Overcoming COVID-19 organization and sites have been previously described.[1,2] Data collected included patient demographics, underlying medical conditions, presenting neurologic signs and symptoms, clinical course, laboratory values, diagnostic findings, treatments, complications, outcomes, and vaccination status. According to the methods in the Overcoming COVID-19 vaccine effectiveness study,[3] patients were categorized as being unvaccinated (no receipt of the BNT162b2 or mRNA-1273 vaccine before illness onset) or vaccinated if the most recent dose (first or second dose of the BNT162b2 or mRNA-1273 vaccine) had been administered at least 14 days before illness onset. Adolescents who had received only one dose of vaccine or who had received a second dose less than 14 days before illness onset were considered to have been partially vaccinated; those who had received two doses at least 14 days before illness onset were considered to have been fully vaccinated.

Reporting guidelines for uncontrolled case series were followed.[4] For all patients with neurologic involvement, the central investigation team composed of experts in pediatric neurology (KLL), pediatric neuroradiology (TYP) and pediatric critical care medicine (AGR)

© 2022 LaRovere KL et al. *JAMA Neurology.*

reviewed the database for demographic information, clinical characteristics, neurologic signs and symptoms, laboratory and cerebrospinal fluid (CSF) results, and findings on clinical reports from brain magnetic resonance imaging (MRI), and brain computed tomography (CT). All cases that could involve severe neurologic involvement were flagged for further review.

Two experts (KLL, AGR) adjudicated local diagnoses for all cases with fatal and "life-threatening" SARS-CoV-2 neurologic conditions. For all deaths and cases with life-threatening neurologic involvement, the experts reviewed additional local source data (e.g. anonymized admission notes, consultant notes, discharge summaries, laboratory and microbiology reports, clinical reports from brain MRI, brain CT, and actual brain MRI and CT images), and personally communicated with site clinicians about each case to confirm neurologic diagnoses and outcomes, discuss the nature of the relationship between neurologic involvement and SARS-CoV-2, and obtain further clinical information when needed. Cases with non-life-threatening neurologic involvement and those without neurologic involvement were selected for formal review by the experts only if there were new neurologic deficits noted at hospital discharge. The experts reviewed local source documents (e.g. evaluations by rehabilitative services) and personally communicated with site clinicians to determine the type of deficits and whether they were most likely directly related to COVID-19 or MIS-C neurologic involvement or sequelae of critical illness (e.g. pneumonia and acute respiratory failure, multi-organ failure) and intensive care therapies (e.g. ECMO, prolonged anesthetic/muscle relaxant use and immobilization).

© 2022 LaRovere KL et al. *JAMA Neurology.*

Standardized case report forms for encephalitis were sent to each site that documented a diagnosis of encephalitis or if an acute imaging abnormality noted in the official report could be suggestive of encephalitis. Cases of acute encephalitis were adjudicated by central experts (KLL, TYP) based upon review of the case report forms, laboratory and EEG data in the database, and findings on neuroimaging studies that were sent to the central site. The diagnosis of encephalitis was classified as 'possible' or 'confirmed' using the International Encephalitis Consortium criteria,[5] which requires altered level of consciousness, lethargy, or personality change lasting ≥24 hours and: 1) ≥2 of the following (possible encephalitis): fever ≥38 degrees Celsius within 72 hours before or after presentation, CSF white blood cell count ≥5/cubic mm, electroencephalography (EEG)/neuroimaging findings consistent with encephalitis not attributable to another cause, focal/generalized seizure not fully attributable to a pre-existing seizure disorder, or new onset focal neurologic findings; or 2) ≥3 features from all of the above (confirmed encephalitis). Patients were included in the Acute Disseminated Encephalomyelitis (ADEM) category if site clinicians indicated a diagnosis of ADEM in the database, the patient was noted to have encephalopathy on the encephalitis case report forms, and imaging reports or actual images had an imaging pattern analogous to ADEM based on expert review. Cerebral venous and sinus thrombosis (CVST) was identified as part of the clinical picture of patients with acute CNS infection/ADEM, and this complication was therefore included in this category.
Stroke was defined as a sudden focal neurologic deficit lasting ≥ 24 hours of presumed vascular origin confirmed on imaging to be caused by infarction (ischemic stroke type) or atraumatic hemorrhage (hemorrhagic stroke type) and correlated with the clinical focal deficit.

© 2022 LaRovere KL et al. *JAMA Neurology.*

All deaths and cases with life-threatening neurologic involvement were classified as related to SARS-CoV-2 neurologic involvement either “directly” or “secondary” to a complication in another organ system (e.g., stroke during extracorporeal membrane oxygenation [ECMO] therapy for heart or lung failure) or exacerbation of an underlying primary neurologic disease based on an understanding of the clinical spectrum of COVID-19 in critically ill children and what is expected during neurocritical illness outside of COVID-19.

Local site principal investigators determined whether patients met CDC criteria for MIS-C. When co-infections with other viruses or bacteria were identified, causality was adjudicated by pediatric infectious disease experts (CVH). Non-CNS organ-system involvement was categorized on the basis of symptoms, clinical findings, and laboratory measures as previously described.[1]

© 2022 LaRovere KL et al. *JAMA Neurology.*

eFigure 1: Eligibility flowchart of hospitalized patients with COVID-19-related neurologic involvement, December 15, 2020 –December 31, 2022

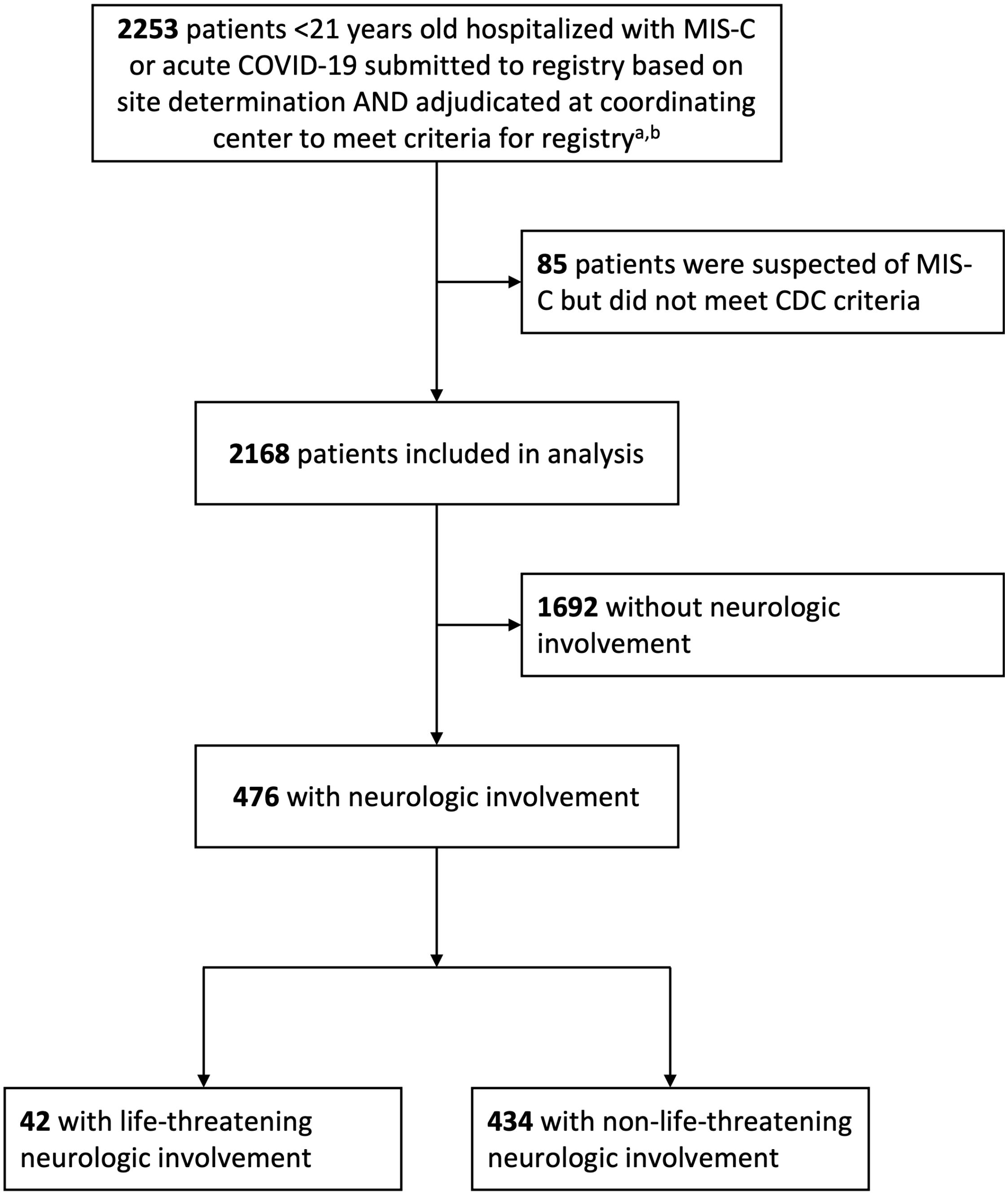

[a]Registry data was based on voluntary case reporting by participating sentinel surveillance sites.
[b]Criteria for registry: meet case definition for MIS-C (**eTable S1**) or evidence of infection with SARS-CoV-2 based on a positive RT-PCR or antigen test within 72 hours of hospitalization for COVID-19-related illness.

© 2022 LaRovere KL et al. *JAMA Neurology.*

eFigure 2. Presenting neurologic symptoms and most abnormal laboratory values in patients (<21 years) hospitalized for COVID-19. **2A.** Presenting neurologic symptoms by age in 476 patients (<21 years) with COVID-19-related neurologic involvement
2B. Most abnormal laboratory results in 2,168 patients (<21 years) with COVID-19 by severity of neurologic involvement. Denominators are provided in **eTable 2** in Supplement.

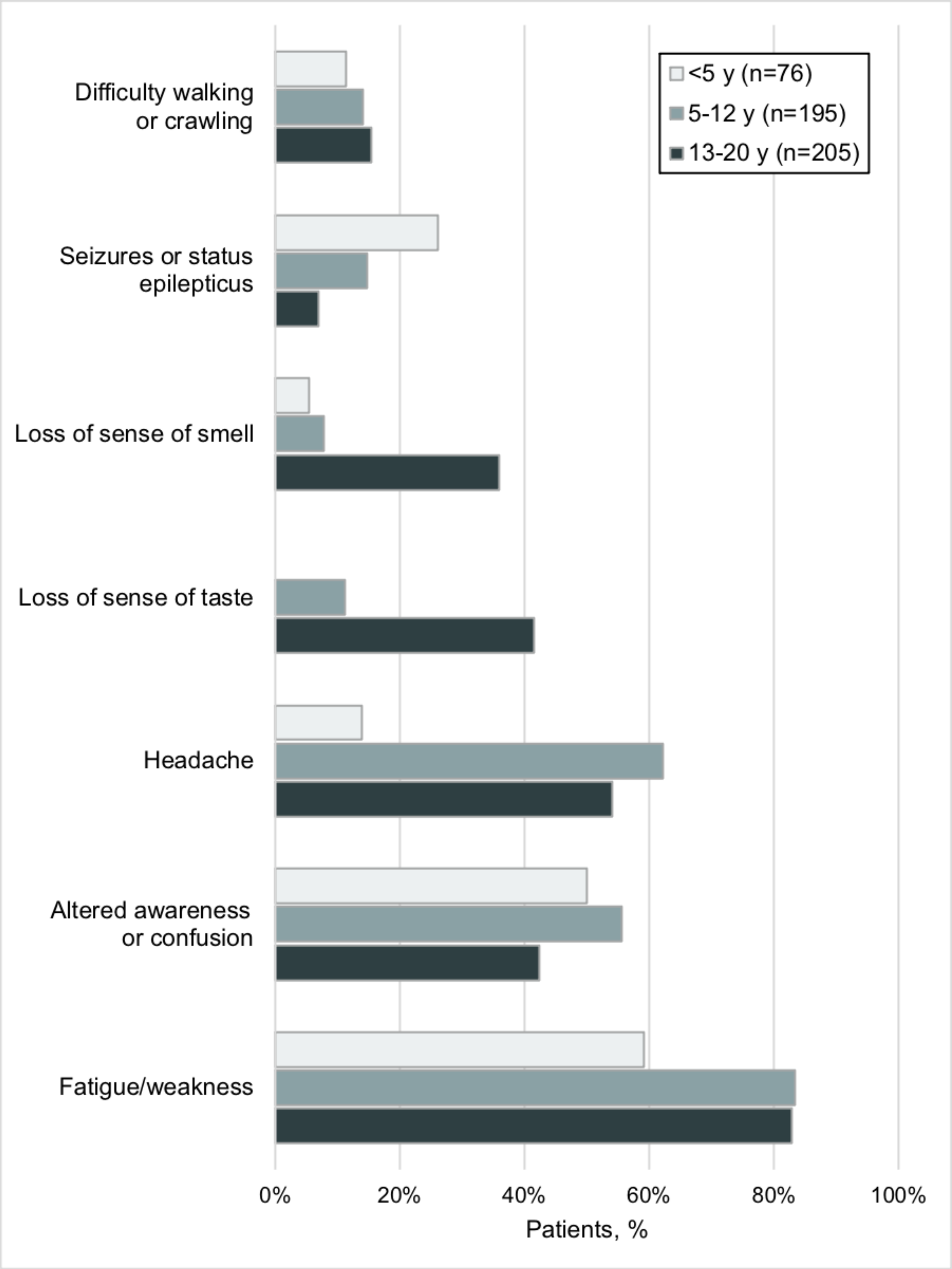

© 2022 LaRovere KL et al. *JAMA Neurology.*

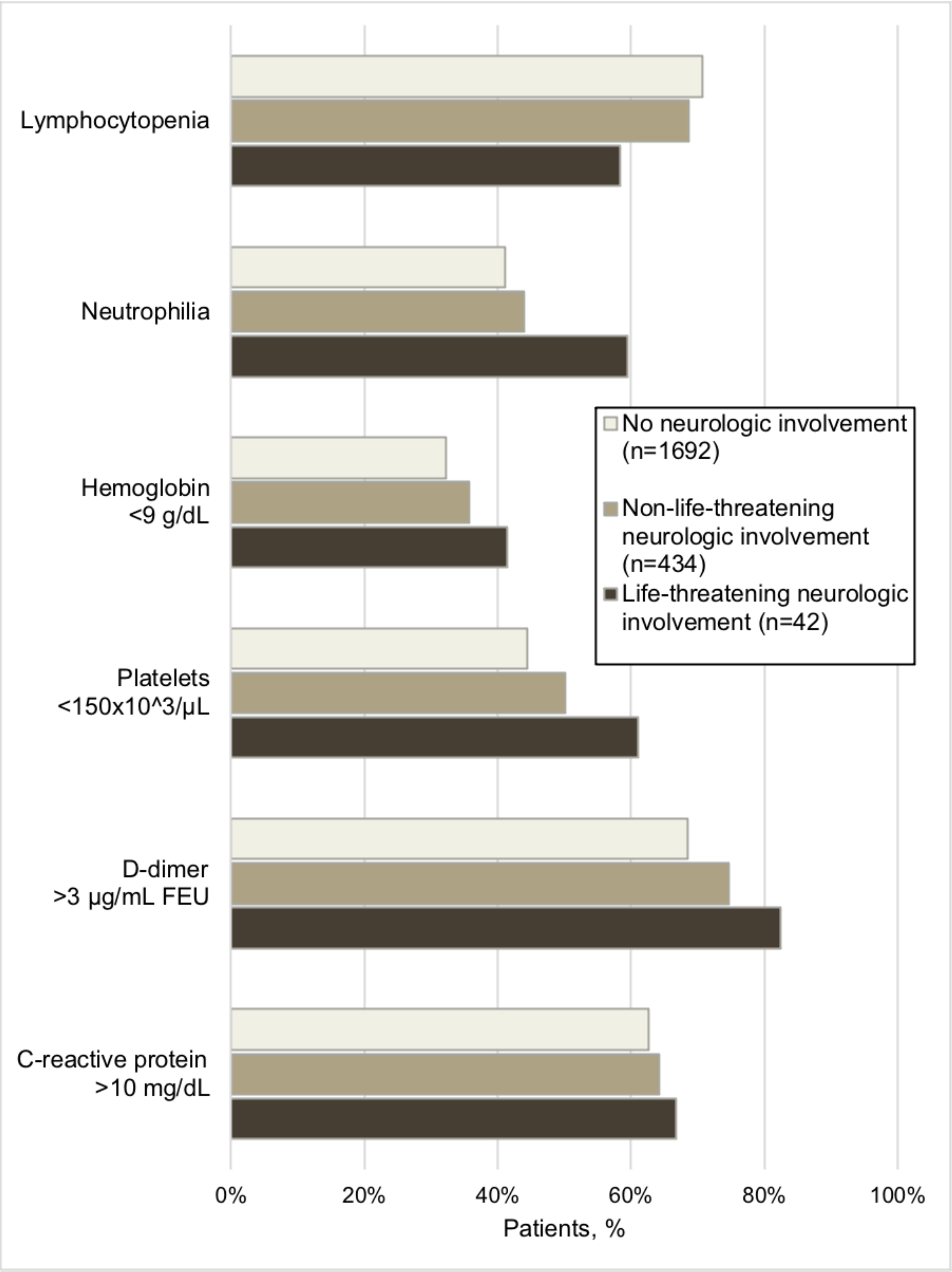

Neutrophilia was defined as a maximum absolute neutrophil count higher than 7700/ μ L. Lymphocytopenia was defined as an absolute lymphocyte count of less than 1500/ μ L in patients 8 months or older and of less than 4500/ μ L in patients younger than 8 months.

© 2022 LaRovere KL et al. *JAMA Neurology.*

eTable 1. Case definition used in this investigation for multisystem inflammatory syndrome in children (MIS-C) developed by the U.S. Centers for Disease Control and Prevention.[6]

- Fever > 38.0°C [a]

AND

- Laboratory evidence of inflammation [b]

AND

- Evidence of clinically severe hospitalized illness among children aged <21 years with multisystem (≥2) organ involvement (cardiac, renal, respiratory, hematologic, gastrointestinal, dermatologic or neurological)

AND

- One of the following:

1. SARS-CoV-2 positive RT-PCR test
2. SARS-CoV-2 positive antibody test
3. SARS-CoV-2 negative RT-PCR and antibody tests but with identified COVID exposure [c] within the four weeks prior to the onset of symptoms

[a] Fever >38.0°C for ≥24 hours, or report of subjective fever lasting ≥24 hours
[b] Including, but not limited to, one or more of the following: neutrophilia; lymphopenia; hypoalbuminemia; and elevated levels of C-reactive protein (CRP), erythrocyte sedimentation rate (ESR), fibrinogen, procalcitonin, d-dimer, ferritin, lactic acid dehydrogenase (LDH), or interleukin 6 (IL-6)
[c] Known exposure to a person with laboratory-confirmed COVID-19 or a clinical diagnosis of COVID-19 within 4 weeks prior to onset of MIS-C.

© 2022 LaRovere KL et al. *JAMA Neurology.*

eTable 2. Number of patients with presenting neurologic symptoms (eFigure 2A) and number of patients with most abnormal laboratory values by severity of neurologic involvement (eFigure 2B) with percentages shown in the Figure.

2A.

**Symptoms**	**<5 years (n=76)**	**5-12 years (n=195)**	**13-20 years (n=205)**
Difficulty walking or crawling	61	163	155
Seizures or status epilepticus	69	176	184
Loss of sense of taste	36	116	125
Loss of sense of smell	37	116	125
Headache	50	172	170
Altered awareness or confusion	68	185	191
Fatigue/weakness	66	180	187

2B.

**Laboratory Values**	**No neurologic involvement (n=1692)**	**Non-life-threatening neurologic involvement (n=434)**	**Life-threatening neurologic involvement (n=42)**
C-reactive protein > 10 mg/dL	1529	377	36
D-dimer >3 μg/mL FEU	1342	320	34
Platelets <150x10^3/μL	1646	419	41
Hemoglobin < 9 d/dL	1649	422	41
Neutrophilia	1497	391	37
Lymphocytopenia	1356	360	36

© 2022 LaRovere KL et al. *JAMA Neurology.*

eTable 3. Life-threatening SARS-CoV-2-related neurologic disorders and outcomes in 42 children and adolescents (<21 years) hospitalized for COVID-19 or MIS-C

Disease (no. cases)	Age Category^	SARS-CoV-2 RT-PCR	SARS-CoV-2 Antibody	MIS-C	Previously Healthy	Underlying Neurologic Disorder	Outcome
**Acute CNS Infection or ADEM (23 cases)**							
Encephalitis	school age	NEG	POS	Yes	No	No	Discharged home
Encephalitis	teen	POS	Not tested	No	No	No	Discharged home
Encephalitis	teen	POS	Not tested	Yes	No	No	New deficits, required acute rehabilitation
Encephalitis	school age	NEG	POS	Yes	No	No	Died‡
Encephalitis	preschool	POS	Not tested	Yes	No	No	Discharged home
Encephalitis	teen	POS	POS	Yes	No	No	Discharged home♦
Encephalitis	teen	POS	POS	No	Yes	No	New deficits, required acute rehabilitation
Encephalitis	school age	NEG	POS	Yes	No	No	Discharged home
Encephalitis	preschool	Not tested	POS	Yes	Yes	No	Discharged home
Encephalitis	school age	POS	POS	No	No	Yes	Discharged home
Encephalitis	infant	POS	POS	Yes	Yes	No	Discharged home
Encephalitis	school age	NEG	POS	Yes	No	No	Discharged home
Encephalitis	teen	POS	NEG	No	No	No	Died‡
Encephalitis	school age	POS	NEG	No	No	No	Discharged home
Encephalitis	school age	NEG	POS	Yes	Yes	No	Discharged home#
Meningitis	toddler	POS	NEG	No	Yes	No	Discharged home
Meningitis	school age	NEG	POS	Yes	No	No	Discharged home
Meningitis	school age	NEG	POS	Yes	No	No	Discharged home
Meningitis	teen	POS	POS	No	Yes	No	Discharged home#
Meningitis	teen	POS	Not tested	No	No	No	New deficits, required acute rehabilitation#
Meningitis	teen	POS	POS	No	Yes	No	Died‡#
Transverse Myelitis	teen	POS	NEG	No	No	No	New deficits, discharged home
Leukoencephalopathy	young adult	POS	Not tested	Yes	Yes	No	Discharged home
**Ischemic or Hemorrhagic Stroke (11 cases)**							
Ischemic	infant	POS	NEG	No	No	No	Died
Ischemic	school age	POS	NEG	No	No	Yes	New deficits, required acute rehabilitation
Hemorrhagic	school age	POS	POS	Yes	No	No	Discharged home

© 2022 LaRovere KL et al. *JAMA Neurology.*

Ischemic	toddler	POS	Not tested	No	No	Yes	Neurologic function at baseline, required acute rehabilitation**
Ischemic	teen	POS	POS	Yes	Yes	No	New deficits, required acute rehabilitation
Hemorrhagic	school age	POS	Not tested	No	Yes	No	New deficits, required acute rehabilitation
Ischemic	toddler	POS	Not tested	No	No	Yes	New deficits, discharged home
Hemorrhagic	infant	POS	POS	No	Yes	No	Died**
Ischemic	teen	POS	Not tested	No	No	No	Died
Ischemic	teen	NEG	POS	Yes	Yes	No	New deficits, required acute rehabilitation
Ischemic	infant	POS	Not tested	No	Yes	No	New deficits, required acute rehabilitation**
**Severe Encephalopathy (5 cases)**							
	teen	NEG	POS	Yes	No	No	Discharged home♦
	school age	NEG	POS	Yes	Yes	No	Discharged home
	teen	NEG	POS	Yes	No	No	Discharged home♦
	teen	POS	POS	No	No	No	Discharged home
	infant	POS	Not tested	No	Yes	No	Discharged home
**Acute Fulminant Cerebral Edema (2 cases)**							
	teen	NEG	POS	Yes	No	Yes	Died‡
	teen	POS	Not tested	No	No	No	Died
**Guillain Barre Syndrome (1 case)**							
	school age	POS	Not tested	No	Yes	No	New deficits, discharged home

Abbreviations: Ab = antibody; ADEM = acute disseminated encephalomyelitis; CNS=central nervous system; GBS = Guillain-Barre Syndrome; MIS-C = Multisystem Inflammatory Syndrome in Children; RT-PCR = reverse transcriptase polymerase chain reaction; POS = positive, NEG = negative, Not tested = test not sent in hospital; CVST = cerebral venous sinus thrombosis

^Age categories: infant < 1 year; toddler 1-2 years; preschool 3-5 years; school age 6-12 years; teen 13-17 years; young adult 18-21 years

**Stroke occurred while on extracorporeal membrane oxygenation (ECMO)

#Cerebral venous and sinus thrombosis was identified as part of the clinical picture

♦Brain MRI with abnormal signal intensity and restricted diffusion in the splenium of the corpus callosum[7]

‡Died by neurologic criteria (brain death)

© 2022 LaRovere KL et al. *JAMA Neurology.*

eTable 4. Detailed clinical descriptions, treatment and outcomes for 15 patients (< 21 years) with clinically adjudicated possible or confirmed acute encephalitis

Case	Age Category^ (Gender)	Timing of Symptom Onset	Neurologic symptoms	CSF WBC count ≥ 5/mm³⁺	EEG⊥	Imaging	Treatment	Hospital LOS (days)	Outcome
**Possible Encephalitis**									
1	school age (male)	4 days PTA	Fever, lethargy, confusion, neck pain/stiffness, difficulty walking, altered speech, memory difficulties	Yes	Not tested	CCT: normal	None	10	Discharged home
2	teen (female)	2 days after admission	Fever, confusion, seizures	No	Electroclinical seizures originating from right occipital region, diffuse background slowing	Brain MRI: increased T2 signal in cingulate gyrus and frontal cortex bilaterally; edema in posterior thalamus, pulvinar region<br>Head MRA: normal	Corticosteroids	17	New deficits, required acute rehabilitation*
3	school age (female)	1 day PTA	Fever, headache, meningismus, confusion, non-sensical speech, seizures	No	Electroclinical seizures originating from left temporal-parietal region, diffuse background slowing	Brain MRI: multifocal regions of reduced diffusivity involving both cerebral hemispheres and deep gray structures with mild diffuse cerebral swelling	ASM	39	DIed‡
4	preschool (female)	6 days PTA	Fever, confusion, lethargy, bilateral lower extremity weakness, abnormal gait	Yes	Not tested	Brain MRI: reduced diffusivity within bilateral external capsules, medial thalami/subthalamic nuclei, corticospinal tracts, bilateral pons, and hippocampi	None	18	Discharged home

© 2022 LaRovere KL et al. *JAMA Neurology.*

5	teen (male)	1 day PTA	Fever, seizure, lethargy and confusion progressing to obtundation, nystagmus	No	Diffuse but frontally predominant sharp waves, diffuse background slowing	NCCT: normal	Corticosteroids	13	Discharged home
6	teen (female)	3 weeks PTA	Fever, headaches, lethargy, confusion, seizure, vomiting, blurry vision, nystagmus	No	Diffuse background slowing	Brain MRI: acute on chronic hydrocephalus due to probable aqueductal stenosis** and T2 prolongation in bilateral cerebellar hemispheres, bilateral thalami and bifrontal white matter and cortex with reduced diffusivity on trace diffusion images	Corticosteroids, IVIG, plasma exchange, Anakinra	124	New deficits, required acute rehabilitation (non-ambulatory, mechanically ventilated by tracheostomy)
7	school age (male)	On day of admission	Fever, lethargy, confusion, hallucinations (tactile), agitation, vomiting	No	Right frontal spike waves, diffuse background slowing	CCT: normal<br>Brain MRI: patchy areas increased T2 and FLAIR signals within parietal deep and subcortical white matter, predominantly on right side	None	7	Discharged home
8	preschool (female)	2 days after admission	Fever, lethargy, confusion, neck pain/stiffness, slurred speech	No	Diffuse background slowing	Brain MRI: normal	Corticosteroids, IVIG, Anakinra	10	Discharged home
9	school age (male)	6 days PTA	Fever, lethargy, confusion, headache, hallucinations, loss of hearing, neck swelling, 6th nerve palsy, areflexia	Not tested	Not tested	Brain MRI: enhancement of the 6th cranial nerves bilaterally[##]	None	17	Discharged home

© 2022 LaRovere KL et al. *JAMA Neurology*.

**Confirmed Encephalitis**									
10	teen (male)	1 day PTA	Fever, worsening lethargy, confusion and agitation, hallucinations (auditory), vomiting, seizures	Yes	Diffuse background slowing	NCCT: Caudate and temporal lobe hypoattenuation<br>Brain MRI♦: increased T2 signal within left medial temporal lobe gyrus	ASM	3	Discharged home
11	school age (female)	2 days PTA	Fever, lethargy, confusion, seizures	Yes	Electrical seizures originating from right hemisphere	Brain MRI: normal	Corticosteroids	3	Discharged home
12	infant (male)	On day of admission	Fever, lethargy, irritability, status epilepticus	Yes	Diffuse background slowing	Brain MRI: increased T2 signal within subcortical white matter of the occipital lobes, bilaterally, and within posterior periatrial and occipital periventricular white matter	Corticosteroids, IVIG, ASMs	5	Discharged home
13	teen (male)	2-3 weeks PTA	Fever, confusion, headache, seizures, orofacial dyskinesias, agitation, slurred speech, difficulty walking, chorea, left sided weakness	Yes[#]	Diffuse background slowing initially; progressed to lack of cerebral electrical activity (iso-electric)	Brain MRI: diffuse edema, innumerable foci of increased T2 signal and reduced diffusivity involving right cerebellum, bilateral frontal, and parietal and occipital lobes especially in superficial and deep white matter; largest single lesion in right cerebellum measuring 2.5. cm in diameter	Corticosteroids, plasma exchange	9	Died‡*

© 2022 LaRovere KL et al. *JAMA Neurology.*

14	school age (female)	5 days PTA	Fever, headache, seizure, neck pain/stiffness, lethargy, confusion progressing to obtundation	Yes	Diffuse background slowing	Brain MRI: cytotoxic lesion of the corpus callosum	None	13	Discharged home
15	school age (male)	1 day PTA	Fever, worsening lethargy, slurred speech, right facial weakness, difficulty walking	Yes	Normal	Brain MRI: diffuse, confluent non-enhancing T2 hyperintensity in the white matter and deep gray nuclei without reduced diffusivity; petechial hemorrhage within corpus callosum; cortical vein thrombosis.	Corticosteroids, IVIG	15	Discharged home

All children had persistent encephalopathy for at least 1 day prior to or on admission.

Abbreviations: ADEM = acute disseminated encephalomyelitis; CNS = central nervous system; GBS = Guillain-Barre Syndrome; MIS-C = Multisystem Inflammatory Syndrome in Children; RT-PCR = reverse transcriptase polymerase chain reaction; PTA = prior to admission; Hospital LOS = length of stay in hospital from admission to discharge; Not tested = test not sent in hospital; CSF = cerebrospinal fluid; EEG = electroencephalogram; CCT = contrast-enhanced cranial computed tomography; NCCT = non-contrast enhanced cranial computed tomography; MRI = magnetic resonance imaging; MRA = magnetic resonance angiography; FLAIR = fluid-attenuated inversion recovery; Treatment = CNS directed therapies provided inpatient for neurological indications; ASM = antiseizure medication; IVIG = intravenous immunoglobulin; Corticosteroids included methylprednisolone, dexamethasone, and/or prednisone

^Age categories: infant < 1 year; toddler 1-2 years; preschool 3-5 years; school age 6-12 years; teen 13-17 years; young adult 18-21 years

[+]CSF white blood cell count ≥5/cubic mm is included in the International Encephalitis Consortium criteria for the diagnosis of encephalitis[5]

[⊥]On electroencephalogram, diffuse background slowing may be suggestive of diffuse cerebral dysfunction (encephalopathy)

♦Had low titer positive MOG antibody (1:20)

*Received influenza vaccine 4-6 weeks PTA

‡Died by neurologic criteria (brain death)

**New diagnosis, patient was previously healthy

[#]Oligoclonal bands present in CSF

[##]This patient with MIS-C had altered consciousness, cranial nerve six palsy and areflexia suggesting overlap between possible Bickerstaff's brainstem encephalitis and Miller Fisher Syndrome.

© 2022 LaRovere KL et al. *JAMA Neurology.*

eTable 5. Vaccination status by SARS-CoV-2-related neurologic involvement and MIS-C versus acute COVID-19 diagnosis

Vaccination Status	No Neurologic Involvement (n=1692)	Non-life-threatening Neurologic Involvement (n=434)	Life-threatening Neurologic Involvement (n=42)	Acute COVID-19 (n=733)	MIS-C (n=1435)
Vaccine Eligible	479 (28)	169 (39)	16 (38)	378 (52)	286 (20)
Fully Vaccinated	7 (1)	3 (2)	1 (6)	6 (2)	5 (2)
Partially Vaccinated	15 (3)	4 (2)	0 (0)	7 (2)	12 (4)
Unvaccinated	407 (85)	141 (83)	15 (94)	328 (87)	235 (82)
Unknown	50 (10)	21 (5)	0 (0)	37 (10)	34 (12)

Abbreviations: MIS-C = Multisystem Inflammatory Syndrome in Children

© 2022 LaRovere KL et al. *JAMA Neurology.*

eReferences

1. Feldstein LR, Rose EB, Horwitz SM, et al. Multisystem Inflammatory Syndrome in U.S. Children and Adolescents. *The New England journal of medicine*. Jul 23 2020;383(4):334-346. doi:10.1056/NEJMoa2021680

2. Feldstein LR, Tenforde MW, Friedman KG, et al. Characteristics and Outcomes of US Children and Adolescents With Multisystem Inflammatory Syndrome in Children (MIS-C) Compared With Severe Acute COVID-19. *Jama*. Mar 16 2021;325(11):1074-1087. doi:10.1001/jama.2021.2091

3. Olson SM, Newhams MM, Halasa NB, et al. Effectiveness of BNT162b2 Vaccine against Critical Covid-19 in Adolescents. *The New England journal of medicine*. Feb 24 2022;386(8):713-723. doi:10.1056/NEJMoa2117995

4. Kempen JH. Appropriate use and reporting of uncontrolled case series in the medical literature. *Am J Ophthalmol*. Jan 2011;151(1):7-10 e1. doi:10.1016/j.ajo.2010.08.047

5. Venkatesan A, Tunkel AR, Bloch KC, et al. Case definitions, diagnostic algorithms, and priorities in encephalitis: consensus statement of the international encephalitis consortium. *Clin Infect Dis*. Oct 2013;57(8):1114-28. doi:10.1093/cid/cit458

6. Centers for Disease Control and Prevention. Information for healthcare providers about multisystem inflammatory syndrome in children (MIS-C). Available at: https://www.cdc.gov/mis-c/hcp/. Accessed on: 4 Dec 2020.

7. Abdel-Mannan O, Eyre M, Lobel U, et al. Neurologic and Radiographic Findings Associated With COVID-19 Infection in Children. *JAMA Neurol*. Nov 1 2020;77(11):1440-1445. doi:10.1001/jamaneurol.2020.2687

© 2022 LaRovere KL et al. *JAMA Neurology*.
