# Supplementary material for: Changes in Distribution of Severe Neurologic Involvement in US Pediatric Inpatients With COVID-19 or Multisystem Inflammatory Syndrome in Children in 2021 vs 2020
Source: JAMA Neurol. 2022 Nov 7;80(1):91–8. doi: 10.1001/jamaneurol.2022.3881 (PMC9641594; doi:10.1001/jamaneurol.2022.3881)
Supplement: Supplement 2. — Nonauthor collaborators [file jamaneurol-e223881-s002.pdf]

Supplemental Online Content: Nonauthor Collaborators
*First name, last name, and suffix (if applicable) are required and will appear in PubMed.

*Group Name: Overcoming COVID-19 Investigators							
***First Name and Middle Initial(s)**	***Last Name**	***Suffix (eg, Jr, III)**	Academic Degrees	Institution	Location (city, state/province, country)	Role or Contribution, eg, chair, principal investigator	Group (if more than 1 Group listed in the byline) and/or Subgroup (eg, Steering Committee)
Mary Glas	Gaspers						
Ronald C	Sanders						
Matt S	Zinter						
Pia S	Pannaraj						
Emily	Port						
Sara	Shankman						
Rachel	Mansour						
Christopher L	Carroll						
Gwenn E	McLaughlin						
Paula S	Espinal						
Kelly N	Michelson						
Bria M	Coates						
Guru	Bhoojhawon						
Janice E	Sullivan						
Vicki L	Montgomery						
Ana Lia	Graciano						
Susan V	Lipton						
Mary Beth F	Son						
Sabrina R	Chen						
Julia	Worden						
Timothy	McCadden						
Ryan W	Carroll						
Phoebe H	Yager						
Neil D	Fernandes						
Janet R	Hume						
Emily R	Levy						
Lacy	Malloch						
Lora	Martin						
Candace	Howard-Claudio						
David	Gourdy						

Supplemental Online Content: Nonauthor Collaborators
*First name, last name, and suffix (if applicable) are required and will appear in PubMed.

***First Name and Middle Initial(s)**	***Last Name**	***Suffix (eg, Jr, III)**	Academic Degrees	Institution	Location (city, state/province, country)	Role or Contribution, eg, chair, principal investigator	Group (if more than 1 Group listed in the byline) and/or Subgroup (eg, Steering Committee)
Philip C	Spinella						
Melissa L	Cullimore						
Russell J	McCulloh						
Lawrence C	Kleinman						
Simon	Li						
Joseph	Kuebler						
Adam J	Ratner						
Heda	Dapul						
Sule	Doymaz						
Michael A	Keenaghan						
Cody	Schwartz						
Steven L	Shein						
Amanda N	Lansell						
Ryan A	Nofziger						
Mary A	Staat						
Neal J	Thomas						
Andrew D	Butler						
Nelson	Reed						
Laura	Stewart						
Meena	Golcha						
Marian	Samperio						
Cindy	Bowens						
Mia	Maamari						
Hillary	Crandall						
Lincoln S	Smith						
John K	McGuire						
Manish M	Patel						
